# Comparing systemic therapy and cognitive behavioral therapy for social anxiety disorders: study protocol for a randomized controlled pilot trial

**DOI:** 10.1186/s13063-016-1252-1

**Published:** 2016-03-31

**Authors:** Christina Hunger, Rebecca Hilzinger, Theresa Koch, Johannes Mander, Anja Sander, Hinrich Bents, Jochen Schweitzer

**Affiliations:** Center for Psychosocial Medicine, Institute for Medical Psychology, Heidelberg University Hospital, Bergheimer Straße 20, D-69115 Heidelberg, Germany; Center for Psychological Psychotherapy, University of Heidelberg, Bergheimer Straße 58a, D-69115 Heidelberg, Germany; Institute of Medical Biometry and Informatics, University of Heidelberg, Im Neuenheimer Feld 305, D-69120 Heidelberg, Germany

**Keywords:** Systemic therapy, Cognitive behavioral therapy, Social anxiety disorder, Adherence, Randomized controlled trial (RCT), Pilot, Manual development

## Abstract

**Background:**

Social anxiety disorders are among the most prevalent anxiety disorders in the general population. The efficacy of cognitive behavioral therapy (CBT) for social anxiety disorders is well demonstrated. However, only three studies point to the efficacy of systemic therapy (ST) in anxiety disorders, and only two of them especially focus on social anxiety disorders. These ST studies either do not use a good comparator but minimal supportive therapy, they do not use a multi-person ST but a combined therapy, or they do not especially focus on social anxiety disorders but mood and anxiety disorders in general. Though ST was approved as evidence based in Germany for a variety of disorders in 2008, evidence did not include anxiety disorders. This is the first pilot study that will investigate multi-person ST, integrating a broad range of systemic methods, specifically for social anxiety disorders and that will compare ST to the "gold standard" CBT.

**Design:**

This article describes the rationale and protocol of a prospective, open, interventive, balanced, bi-centric, pilot randomized controlled trial (RCT). A total of 32 patients with a primary SCID diagnosis of social anxiety disorder will be randomized to either CBT or ST. Both treatments will be manualized.

The primary outcome will include social anxiety symptoms at the end of therapy. Therapy will be restricted to no more than 26 hours (primary endpoint). Secondary outcomes will include psychological, social systems and interpersonal functioning, symptom adjustment, and caregiver burden, in addition to change measures, therapist variables and treatment adherence. At the secondary endpoints, 9 and 12 months after the beginning of therapy, we will again assess all outcomes.

**Discussion:**

The study is expected to pilot test a RCT which will be the first to directly compare CBT and multi-person ST, integrating a broad range of systemic methods, for social anxiety disorders, and it will provide empirical evidence for the calculation of the number of patients needed for a confirmatory RCT.

**Trial registration:**

ClinicalTrials.gov: NCT02360033; date of registration: 21 January 2015.

## Background

Both cognitive behavioral therapy (CBT) and systemic therapy (ST) have a long tradition in the psychotherapeutic treatment of various disorders. However, CBT has a much stronger history of manualization and evaluation, especially for patients with anxiety disorders [1]. It is also part of insurance-funded German psychotherapy, which ST is not. ST was approved as evidence based in Germany for a variety of disorders, but evidence did not include anxiety disorders [2, 3]. Three randomized controlled trials (RCTs) for anxiety disorders are available [4–6]. However, they either do not especially focus on social anxiety disorders but mood and anxiety disorders in general [4], they do not use a multi-person ST but a combined therapy [5], or they do not use a "gold standard" comparator but minimal supportive therapy (MST) [6]. We therefore need more studies that investigate the efficacy of ST specifically for social anxiety disorders, and that use a multi-person ST compared to a well-investigated comparator, such as CBT.

### Epidemiology

Social anxiety disorders are among the most prevalent anxiety disorders (prevalence of 7–16 %). In general, women are affected more frequently (3:2) [7]. In clinical populations, we find women and men in equal shares [8]. Both women and men are affected by the many significant psychosocial and occupational limitations that accompany social anxiety disorders [9]. Those affected have lower income and education, higher unemployment, are more often unmarried and have fewer friends [10]. The age of onset of social anxiety disorders averages 10–13 years [7]; spontaneous remission in the first 2 years is below 20 % [11, 12].

### Clinical picture

Social anxiety disorders are interpersonal disorders. Symptoms of fear such as shaking or flushing arise when the affected person experiences that he or she may attract attention, and that symptoms such as shaking or flushing constrain his or her ability to build up social relationships [13]. Socially anxious individuals display little emotionality, intimacy and secure bonding [14]. The performance anxiety type describes, for example, fears being seized with stage fright, behaving awkwardly if aware of being scrutinized by others and/or getting flurried in larger groups when speaking publicly or eating in restaurants. The interpersonally anxiety type describes, for example, fear of giving an impression of being a bore, an odd fellow and/or difficult to get along with so that the individual is the only one out of keeping with company in everyday situations in medium-sized groups, for example, during lunch with work colleagues or at a garden party with neighbors [15]. People with the specific subtype fear to speak or to eat in public in some specific social situations. Individuals with the generalized subtype fear such behavior in a greater number of situations. The generalized subtype shows stronger burden caused by the generalized socially anxious symptomatology and by more severe comorbidity [7].

### Psychotherapy

CBT explains the disposition of social anxiety disorders by cognitive schemas which stimulate misleading internal information processing. Socially anxious individuals show increased self-focused attention when interacting with others stimulated by the assumption that others can see their anxiety. Based on a linked reduction in observation of other people, they focus on negative reactions and tend to interpret ambiguous and neutral feedback in a negative way. This results in excessively negative inferences about how they appear to others. Incorrect cognitions induce avoidance of social situations and/or the extensive use of safety behavior (e.g. avoiding eye contact, speaking low). The intention is on the prevention of feared catastrophes. However, avoidance and safety behavior contributes to the maintenance of negative beliefs and the increase of feared symptoms. They make patients come across to others in ways that are likely to elicit less friendly responses [8, 16, 17].

CBT aims to reverse the maintaining processes specified in the cognitive model of Clark and Wells [17]. The goal is to establish a realistic self-perception. The CBT manual [8] differentiates between five phases which are described in detail in the method section.

ST explains the development of social anxiety disorders by reciprocal interpersonal interactions. Symptoms of social anxiety serve the near-distance regulation when interacting with others. They represent the (non-verbal) communication of dyadic or multi-person “between-us” quality of relationship in private (e.g. family, couple, friends) or professional (e.g. work colleagues, superior-inferior) social systems. Social anxiety indicates that an actual development task has not yet been accomplished and that socially anxious behavior, feelings and thoughts appear to be the best solution currently available. ST thus interprets social anxiety as an individual’s sensitive reaction when fearing being scrutinized and socially rejected. However, maintaining social system structures reciprocally maintains social anxiety [18, 19].

As we did not find a multi-person ST manual for treating specifically social anxiety disorders, JS and CH developed the first manualization of ST, integrating a broad range of systemic methods, for this type of disorder. We reviewed ST manuals for social anxiety disorders in child and adolescent psychotherapy [19] and well-established ST manuals for different disorders [20–22]. We used general ST concepts [18], integrating constructivist, solution-oriented [5, 23] and strategic [24–27] methods, in addition to attending disorder-specific relational ST dynamics [19, 28, 29]. According to the literature and our experiences of treating patients, the aim of ST is to contextualize symptoms of social anxiety by addressing an individual’s important private and/or professional social system. Social anxiety disorders indicate the disturbance of an entire social system and most if not all of its members. It does not rely on one single individual only. Consequently, the primary goal of ST is to identify and involve all important social system members. Thus, they will be invited to therapy sessions in addition to the individual patient. If they cannot attend physically, circular questions serve their inclusion into the room on a cognitive level. The analysis of transgenerational relationships, of past and present interpersonal interactions serve the development of a new understanding of the important roles, places and resources of all system members.

The systemic model of Schweitzer and Hunger [30] combines three different therapy settings with different participants in a multisystemic therapy concept. The first two therapies, and the majority of all therapy sessions alike, are individual but with a strong focus on relationship issues. They are combined with sessions with partners, parents or closest friends, and with one 3-hour group therapy session bringing together project patients and therapists. The ST manual differentiates between four phases which are described in detail in the method section.

### Efficacy

The efficacy of CBT for social anxiety disorders is well established [31]. A meta-analysis of 29 RCT studies showed a general effect size of 0.70 [32]. The largest German multicenter RCT investigating social anxiety disorders is the Social Phobia Psychotherapy Network (SOPHO-NET; *n* = 495) [18]. The SOPHO-NET was initiated by the lack of evidence for the efficacy of a new psychotherapy method for social anxiety disorders. In the SOPHO-NET, this was psychodynamic therapy (PD) which was compared to CBT. Based on the Liebowitz Social Anxiety Scale (LSAS), the authors investigated remission (LSAS score ≤30) and response (LSAS score reduction of at least 31 %) demonstrably comparable to an improvement of ≤2 in the Clinical Global Impression (CGI) [33]. The authors expected superiority of CBT with a small effect (Cohen’s *h* = 0.30; i.e. CBT response rate of 70 %, PD response rate of 55 %) [34]. With smaller effect than expected, results demonstrated superiority for remission (36 % CBT, 26 % PD; *h* = 0.22) but not for response (60 % CBT, 52 % PD) [35]. Again with small effect sizes, secondary interpersonal outcome measures such as the Inventory of Interpersonal Problems (IIP) [36] also showed significant differences in favor of CBT. At 6, 12 and 24 months after the end of treatment, significant between-group differences were no longer found in any outcome. Contrariwise, some other studies found that CBT patients tend to continue with problems in relationship formation after the end of therapy [37, 38].

Considering ST, we found only three RCTs for anxiety disorders [4–6], and only two of them investigated social anxiety disorders. One RCT (*n* = 120, Poland) compared 10 weeks of brief strategic therapy (BST) with MST. The outcome relied on the Interpersonal Sensitivity, Anxiety and Global Severity Index of the Symptom Checklist-90-R (SCL-90-R) [39, 40]. This trial demonstrated BST superiority for patients with social anxiety disorders only (response: 50 % BST, 20 % MST) but no significance of any treatment for patients with comorbid personality disorders (response: 7 % BST and MST) [6]. The second RCT (*n* = 83, Germany) compared a combined resource-oriented cognitive-behavioral therapy (ROCBT) with CBT. The main outcomes were the Social Interaction Anxiety Scale (SIAS) and Social Phobia Scale (SPS). SIAS and SPS are original German scales and were developed by the leading German CBT researcher for social anxiety disorders [41–43]. This trial demonstrated ROCBT superiority (Cohen’s *d* = 1.29, for both SIAS and SPS) compared to CBT (*d* = 0.86, for SIAS; *d* = 0.97, for SPS). Therapy effects were stable over the 2- and 10-year follow-up in both treatment conditions [5]. The third RCT (*n* = 326, Finland) compared long- and short-term PD with solution-focused ST. This study included patients with both mood and anxiety disorders. Results indicated a statistically significant reduction of symptoms on all outcomes in all three treatment groups during the 3-year study period, including the SCL-90-R anxiety subscale and the Hamilton Anxiety Rating Scale (HAM-A). Both instruments measure anxiety but not social anxiety [44]. The reduction was faster in short-term psychotherapies, including the solution-focused ST during the first year of follow-up. However, the reduction continued during the 3-year follow-up only for the long-term PD [4].

Consequently, none of the above described ST RCTs used the LSAS, though it is most often used for the assessment of social anxiety disorders [45]. The SOPHO-NET used the LSAS as the main outcome for that reason [35]. Willutzki and colleagues decided to use the original German SIAS and SPS, which highly correlate with the LSAS [5]. We therefore miss information regarding the efficacy of multi-person ST on established social anxiety assessments, such as the LSAS, SIAS and SPS. Additionally, we also miss information regarding the efficacy of multi-person ST when compared to a well-established comparator, the CBT.

### Pilot study

RCTs are regarded as the “gold standard” of present day evidence-based research in psychotherapy. They are often complex, time-consuming and expensive. Before a large RCT is undertaken, a pilot study should be conducted that mimics all the major essentials of the planned larger study [46]. As this study is the first trial that includes both a multi-person ST, integrating a broad range of systemic methods, and CBT, especially focusing on social anxiety disorders, we will have to conduct this pilot RCT before planning in detail the future main trial. Due to the mismatch of this study with previous investigations of ST for social anxiety disorders, reliable information that is needed for the sample size calculation for the main RCT is missing. Thus, this pilot study will help us to pay careful attention to sample size calculation, it will aim to save costs and use patients efficiently in the future RCT [47].

## Hypotheses

The main focus of this paper is to answer the question of what effect sizes of ST can be expected compared to CBT, to calculate how many patients will be needed to replicate effects indicated in this study. For that purpose, we descriptively and exploratively analyze the following hypotheses.

- **H1: Effects for social anxiety at the end of therapy (primary endpoint).** According to the SOPHO-NET [36] and the study by Willutzki and colleagues [5], we expect both CBT and ST to demonstrate a reduction of social anxiety symptoms, and that CBT will show a major change compared to the new ST.
- **H2: Effects for secondary outcomes at the end of therapy (primary endpoint).** According to the SOPHO-NET [36], we expect CBT to demonstrate a major change compared to the new ST on psychological functioning, social system and interpersonal functioning, symptom adjustment, and caregiver burden.
- **H3: Effects 9 and 12 months after the beginning of therapy (secondary and tertiary endpoint).** According to the SOPHO-NET [36] and the study by Knekt and colleagues [4], we expect both CBT and ST to show stable effects on primary and secondary outcomes during the follow-up period.

## Methods

### Design and sample size

**Fig. 1:**
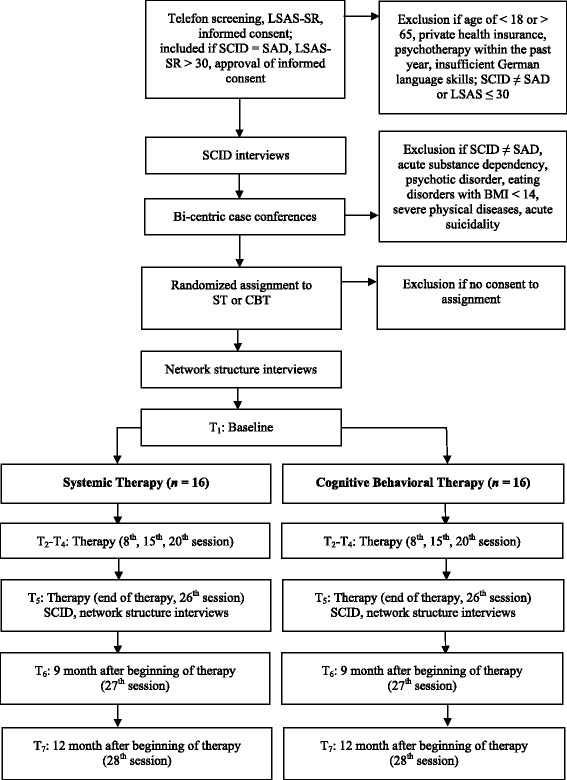
Design, assessments and patient flow (CONSORT chart)

This pilot study is a prospective, open, interventive, balanced, bi-centric^1^, explorative RCT. The aim is to compare CBT and ST for social anxiety disorders. According to Cocks and Torgerson [47] for two-arm pilot studies of this kind, we will recruit 32 patients; 16 patients per CBT and ST, respectively (Fig. 1). Cocks and Torgerson used a confidence interval (CI) approach to calculate pilot sample sizes for continuous outcome measures. They supposed 0.3 of a standard deviation between two groups to be worthwhile, and stated that such a study would require 350 participants (assuming 80 % power and a two-sided alpha of 5 %). For pilot studies of which the primary goal is testing of the recruitment rate, they found 32 patients (approximately 9 % of the main sample size) to be required to produce a one-sided 80 % confidence limit. Cocks and Torgerson concluded that a pilot trial with that sample size, showed an estimate larger than zero and demonstrated feasibility to recruit and retain the patients, and so forth, should move forward with the main study.

### Ethical consideration

CBT will be practiced at the Center for Psychological Psychotherapy (CPP), Heidelberg University, and ST at the Institute of Medical Psychology (IMP), Heidelberg University Hospital. All therapies will be recorded. The pilot study design, the measurement times and instruments are aligned with the studies of Willutzki and colleagues [5], Rakowska and the SOPHO-NET [48]. This research was approved by the Ethics Committee of the Heidelberg Medical Faculty (S-190/2014). Written informed consent will be obtained from each interested person. Staff will send the study information, informed consent and declaration about the usage of DVD records to each interested person after the initial telephone interview. The staff will be available for any questions on the telephone or face-to-face at the IMP during workdays. All interested persons will be informed about their rights to end their participation at any time without negative consequences. Confidentiality will be maintained at all levels of the pilot study by staff members, diagnosticians, therapists, supervisors and researchers. All of them have to declare bindingly that they will give no information to third persons. Additionally, we will work with identification codes for each interested person and patient. All data will be saved pseudonymized on the IMP server. This pilot trial will be conducted in accordance with the Declaration of Helsinki [49], as well as with the State Chamber of Physicians of Baden-Wuerttemberg, Germany.

### Recruitment

#### Patients

Recruitment started in 2014, with the first patient included on 18 November 2014, and we expect to complete recruitment in spring 2016. We distributed flyers and study information to doctors, psychological psychotherapists, occupational therapists, hospitals and psychosocial counseling centers in and around Heidelberg; gave public announcements in the local press, on the IMP website, information portals on clinical trials and Facebook; mailed to the Heidelberg University, the Heidelberg University Hospital and universities near Heidelberg; and we gave our flyer and study information to the outpatient centers of the CPP, IMP and the Central Institute of Mental Health in Mannheim. All patients will present themselves to the study team and no patients will be referred.

#### Therapists

Recruitment started in 2014, with the first therapists included in September, and was completed in November of the same year. A total of 16 therapists (eight CBT therapists; eight ST therapists) will be involved to conduct two therapies each. CBT therapists will be psychologists in their second to fourth year of training at the CPP. ST therapists will be third- to fourth-year trainees from the Helm Stierlin Institut (HSI), Heidelberg, and the Mannheim Society for Systemic Therapy (MAGST), Mannheim; most of them will be psychologists and educators. Due to the fact that CBT and ST have very different training structures, contents and certification procedures, we chose advanced therapists with different years of training in CBT and ST, respectively, of whom we expected best comparison regarding their training status in theory and practice.

### Study procedures

#### Screening and diagnostic process

Interested individuals will initially take part in a telephone screening assessment of the main socially anxious symptoms (SCID, social anxiety disorder). Persons meeting the inclusion criteria will then receive the LSAS-SR self-report measure and SCID screening questionnaires together with the study information, informed consent and declaration about the DVD record use by post. After signing and sending back these documents, the documentation will be checked and the LSAS-SR score will be calculated and analyzed. If all documents are signed and the interested person shows a LSAS-SR >30, then the individual will be invited to a SCID-I and -II face-to-face interview, including the assessment of the general level of functioning (Global Assessment of Functioning; GAF). These pre-SCID interviews will be rated by independent raters with respect to both primary and secondary diagnoses. After inclusion based on shared decision making by clinical experts in the bi-centric case conference, persons will be randomized. Subsequently, they will be informed whether they will be treated in the CBT or ST study arm. Individuals will then attend a social network interview. In this interview they will select a significant other (e.g. partner, family member, best friend) who will then be asked to fill in a questionnaire booklet assessing significant others' burden.

##### Therapy (T1 to T5)

We will assess study outcomes before the start of therapy, after the 8th, 15th and 20th hour of therapy, as well as after therapy has ended. At each time of assessment, patients will participate in an online survey (UNIPARK) including study outcomes (Table 1). The assessment will be conducted at the IMP. Baseline data (T1) will be scheduled no more than 5 days before the first therapy session. Therapy process (T2 to T4) will be assessed no more than 5 days after the 8th, 15th and 20th hour of therapy. The primary endpoint (T5) will be defined by a joint patient-therapist decision to end therapy or by the end of 26 hours of therapy. All outcome variables will be assessed anew, along with in- and outpatient health care service data. A post-SCID and social network interview will also be conducted. Like the pre-SCID, the post-SCID interviews will be rated by independent raters considering primary and secondary diagnoses.

**Table 1:** Assessment measures and application plan

Purpose	Perspective	Domain	Instrument	Screening	Baseline	8th, 15th, 20th hour of therapy	End of therapy, no later than 26th hour of therapy	9 and 12 months after beginning of therapy
					T1	T2 to T4	T5	T6 and T7
Screening	Pat	Social anxiety	LSAS-SR	X				
	Diag	Mental disorder SAD	SCID: SAD	X				
Outcome	Diag	Mental disorders	SCID I, II	X			X	
	Pat	Social anxiety	LSAS-SR	X	X	X	X	X
	Pat	Social anxiety	SIAS	X	X	X	X	X
	Pat	Social anxiety	SPS	X	X	X	X	X
	Diag	Global functioning	GAF	X	X		X	
	Pat	Depression	BDI-II	X	X	X	X	X
	Pat, Ref	General symptom severity	BSI	X	X	X	X	X
	Pat, Ref	Experience of social systems	EXIS	X	X	X	X	X
	Pat, Ref	Evaluation of social systems	EVOS	X	X	X	X	X
	Pat, Ref	Dyadic adjustment	DAS-12	X	X	X	X	X
	Pat	Interpersonal problems	IIP-32	X	X	X	X	X
	Pat, Ref	Symptom adjustment	ASS	X	X	X	X	X
	Ref	Burden	BAS		X	X	X	X
Process	Pat, T	Therapeutic alliance	SACiP			X	X	X
	Pat, T	Therapeutic relationship	SRS			X	X	X
	Pat	Therapy motivation	URICA			X	X	X
	Pat, T	Credibility	OAT		X			
Therapists	T	Common therapist factors	DPCCQ		X	X	X	X
Quality	R	Adherence and competence	STAS, CTAS-SP		X	X	X	X
Additional service	Pat	Service receipt	CSSRI-EU				X	X

Diag, diagnostician; Pat, patient; R, researcher; Ref, reference person (most important significant other); T, therapist. ASS, Adjustment to Symptomatology Scale; BAS, Burden Assessment Scale; BDI-II, Beck Depression Inventory II; BSI, Brief Symptom Inventory; CSSRI-EU, Client Sociodemographic and Service Receipt Inventory; CTAS-SP, Cognitive Therapy Adherence Scale for Social Phobia; DAS-12, Dyadic Adjustment Scale; DPCCQ, Development of Psychotherapist Common Core Questionnaire; EVOS, Evaluation of Social Systems; EXIS, Experience in Social Systems; GAF, Global Assessment of Functioning; IIP-32, Inventory of Interpersonal Problems, short form; LSAS-SR, Liebowitz Social Anxiety Scale, self-report measure; OAT, Opinion About Treatment; SACiP, Scale for the Multiperspective Assessment of General Change Mechanisms in Psychotherapy; SCID I,II, Structured Clinical Interview for DSM Disorders I and II; SCID: SAD, Structured Clinical Interview for DSM disorders: Social Anxiety Disorders; SIAS, Social Interaction Anxiety Scale; SPS, Social Phobia Scale; SRS, Session Rating Scale; STAS, Systemic Therapy Adherence Scale; URICA, University of Rhode Island Change Assessment Scale

##### Follow-up (T6 and T7)

We will assess patients at 9 and 12 months after the beginning of therapy. Again, patients will participate in the online survey including the outcomes (Table 1). The assessment will be conducted at the IMP. The study will close 12 months after the beginning of therapy of the last included patient.

### Inclusion and exclusion criteria

#### Patients

Inclusion criteria require patients: 1) to be primarily diagnosed with a social anxiety disorder (SCID [50]; corresponding ICD diagnosis: F40.1); 2) to have a LSAS-SR total score >30; 3) to be on stable medication for at least 4 weeks, in cases of concurrent pharmaceutical treatment; 4) to agree to participate in the pilot study and to be randomized into one of the study arms; 5) to agree to not use additional therapeutic support during the study period; and 6) to be 18–65 years old. Exclusion criteria are as follows: 1) acute substance dependency; 2) psychotic disorders; 3) eating disorders (BMI <14); 4) severe physical diseases; 5) acute suicidality; 6) insufficient German language skills; and 7) private health insurance and participation in psychotherapy financed by the national health insurance companies within the past year due to administrative regulations at one of the centers. Comorbid disorders are allowed as long as the social anxiety disorder is of primary concern.

#### Therapists

Inclusion criteria required therapists: 1) to be advanced trainees with more than 300 hours of theoretical and clinical training; 2) to have therapeutic self-experience of at least 100 hours; 3) to have completed an internship in psychosomatic medicine, psychotherapy or psychiatry of at least 600 hours; and 4) to participate in the CBT or ST training and supervisions. If therapists have training in both approaches, they will be assigned to the approach that they consider their primary therapeutic identity. We will choose advanced trainees due to their strong treatment adherence and ability to deliver treatments effectively in outpatient settings [51].

### Randomization

Following inclusion, patients will be randomized, using block randomization in a 1:1 ratio to obtain equal group sizes [52]. We will use a randomization plan generator (www.randomization.com). An independent research team will conduct the assignment of patients to study arms. Therapists will be randomly assigned to patients as they enter the project.

### Blinding and allegiance

#### Blinding

Patients will be informed about their assignment (no blinding, open trial), the general study aims and procedures, but not about specific hypotheses. Blinding will not be feasible since both the CPP and IMP are well known for their specific therapy orientation, and because of the focus on the external validity and generality of the pilot study to routine care [53]. Outcome assessments at all points in time will be conducted online (UNIPARK), and no blinding of assessors will be needed. Pre- and post-SCID diagnostics will be blinded and the assessors will not know the study arm in which each patient will be treated.

#### Allegiance

The preference of researchers and therapists for a particular treatment may account for bias in outcome data [54]. To avoid such bias, we will balance direct and indirect allegiance by a bi-centric trail. In this trail, CBT and ST trainers, supervisors and therapists will be distinct persons but equally allegiant to their method. They will be well trained, follow equally elaborate but quite distinctive manuals. They will receive equal support, and contribute an equal number of hours and days to the project [55].

### Intervention

#### Manuals

The CBT manual [8] is well established. The goal is to establish a realistic self-perception. It includes: 1) generation of an idiosyncratic version of the disorder and identification of individual safety behaviors; 2) manipulation of self-focused attention and safety behaviors in role plays, and using video feedback to demonstrate their adverse effects; 3) training in attentional redeployment and reduction of safety behaviors through behavioral experiments (expositions), cognitive restructuring and changing of dysfunctional convictions; 4) relapse prevention; 5) booster sessions after the end of therapy to refresh therapeutic gains.

The ST manual was written by JS and CH, with consultations from Dr Rüdger Retzlaff (Heidelberg University Hospital). It is the first manual that reviewed ST manuals for social anxiety disorders in child and adolescent psychotherapy [19] and well-established ST manuals for different disorders [20–22], in addition to the well-written CBT and PD manuals for social anxiety disorders [8, 56, 57]. It is based on general ST concepts [18], integrating constructivist, solution-oriented [5, 23] and strategic [24–27] methods, in addition to attending disorder-specific relational ST dynamics [19, 28, 29]. JS and CH tested the manual with two patients before teaching it to the study therapists. Training experiences were utilized for a last revision before study therapies started. According to this manual [30], ST combines three different therapy settings with different participants in a multisystemic therapy concept. The first two therapies, and the majority of all therapy sessions alike, are individual but with a strong focus on relationship issues. They are combined with sessions with partners, parents or closest friends, and with one 3-hour group therapy session bringing together project patients and therapists. The ST manual differentiates between four phases: 1) generation of an idiosyncratic version of the function of the social anxiety symptoms including the identification and inclusion of important private and/or professional social systems and the important members within these systems; 2) experimentation with changes through symptom prescription, paradoxical intention, externalization, deconstruction of shared belief systems and enactment of socially anxious interactions with and without important social system members; 3) relapse prevention involving important social system members; and 4) booster sessions after the end of therapy including important social system members to gain a shared refreshment of therapeutic gains.

#### Training and supervision

All therapists participated in a 3-day CBT or ST training. Dr Denise Ginzburg (Institute of Psychology, Johann Wolfgang Goethe University of Frankfurt, Germany) conducted the CBT workshops; JS and CH conducted the ST workshops. Supervisions were obligatory for one out of four therapy hours. Eva Vogel and Dr Meike Peters (CPP) supervised the CBT group; JS supervised the ST group.

#### Adherence

Therapists will estimate their adherence after each session. Independent raters will rate therapy session video recordings for manual adherence. We are currently developing a Systemic Therapy Adherence Scale (STAS) and will utilize the Cognitive Therapy Adherence Scale for Social Phobia (CTACS) [58] as well.

### Participation and attrition, responses to crises and suicidality

We will measure participation and attrition by counting the number of interested persons who will 1) contact the recruiting office, 2) participate in the initial screening, 3) will be SCID-interviewed, 4) included into the pilot study, 5) drop out before the beginning of therapy, 6) start therapy, 7) drop out during therapy, 8) end therapy, 9) participate in the 9 months follow-up, and 10) participate in the 12 months follow-up. We will describe causes for withdrawing, and calculate the retention rate for both study conditions separately and together.

Patients will be instructed to contact the therapist and/or research team whenever experiencing adverse events. No constraints will exist regarding additional therapeutic consultations. Inclusion criteria will, however, not allow for use of additional counseling or therapeutic support during the study period.

### Measures

#### Screening and assessments

In the screening phase, interested persons will receive pilot study information, will be asked for the fulfillment of inclusion and exclusion criteria, and will be SCID-interviewed for a social anxiety disorder. Potential patients will receive the LSAS-SR and screening questions for the SCID interview. In bi-centric case conferences, supervising experts of both the CPP and IMP, together with the SCID-diagnosticians, will discuss whether the cases will fulfil the inclusion criteria.

##### Primary outcome measures: social anxiety

The 24-item LSAS-SR assesses social interaction and performance situations feared and the degree of avoidance in the past week [59, 60] with excellent internal consistency [61] and good sensitivity to change [45]. The 20-item SIAS and SPS assess social anxiety in interpersonal interactions (SIAS) or while being observed by others (SPS) with good to excellent internal consistencies and sensitivity to change [41–43].

##### Secondary outcome measures: psychological functioning

The GAF scale measures clinicians’ evaluation of patients’ psychosocial functioning [62, 63] with high levels of interrater reliability [64] and sensitivity to change [62]. The 21-item Beck Depression Inventory II (BDI-II) detects cognitive, motivational, affective and somatic symptoms of depression with excellent to good internal consistencies [65, 66] and high sensitivity to change [67]. The Brief Symptom Inventory (BSI) assesses a variety of mental symptoms, and is highly correlated to the original SCL-90-R [68–70].

##### Social system and interpersonal functioning

The 12-item Experience in Social Systems Questionnaire (EXIS) assesses patients' experience within their important social systems. The EXIS shows excellent to satisfactory internal consistencies and good sensitivity to change [71]. The 12-item Dyadic Adjustment Scale (DAS-12) measures the quality of marital relationships showing satisfactory internal consistency [72]. We apply the DAS-12 not only to romantic or marital partners, but also to other caregivers (e.g. family member, best friend). The 32-item Inventory of Interpersonal Problems (IIP-32) identifies a person’s interpersonal difficulties revealing excellent to satisfactory internal consistencies [36, 73] and satisfactory sensitivity to change [74].

##### Symptom adjustment

We developed a 5-item Adjustment to Symptomatology Scale (ASS), inspired by the Couples Interaction Checklist (CIC) [75], but with a focus on a patient’s adjustment to psychological problems in terms of social interactions between the patient and significant others. We are currently investigating its psychometric structure in a separate research project [76].

##### Caregiver burden

The 19-item Burden Assessment Scale (BAS) measures burden by intimate others with excellent to good internal consistencies and satisfactory sensitivity to change [77]. Our German validation found the BAS psychometric reliable with good internal consistencies [78].

##### Change measures

The 21-item Scale for the Multiperspective Assessment of General Change Mechanisms in Psychotherapy (SACiP) measures general change mechanisms with excellent to satisfactory internal consistencies and demonstrated validity by predicting outcome [79, 80]. The 4-item Session Rating Scale (SRS) measures therapeutic alliance [81], and its validity is confirmed by positive correlations with outcome [82]. The 16-item University of Rhode Island Change Assessment Scale (URICA) measures stages of change with excellent to satisfactory internal consistencies and demonstrated validity by predicting symptoms [83].

##### Credibility

The Opinion About Treatment (OAT) questionnaire, adapted from Borkovec and Nau’s (1972) credibility and expectancy questionnaire, assesses how successfully patients think the treatment will be and appears with excellent internal consistencies [84]. We will adapt the OAT to a therapist version as well.

##### Therapist variables

The Development of Psychotherapist Common Core Questionnaire (DPCCQ) characterizes therapists on several subscales [85], of which we chose interpersonal style, relational skills, quality of therapists’ personal lives and difficulties in practice to be suitable for this pilot study. The scales have been found predictive of the therapeutic alliance and outcome [86–88].

##### Service receipt

The Client Sociodemographic and Service Receipt Inventory (CSSRI-EU) can be used for the calculation of costs for health service utilization and medication [89]. Face validity and cross-cultural adaptation are achieved [90].

### Statistical analyses

All statistics will be descriptive and explorative using the SPSS statistical package (version 19.0; IBM, Frankfurt, Germany). In addition to descriptive measures such as mean, standard deviation, median, interquartile range and confidence intervals, comparisons of primary endpoints between the two groups will be performed using t-test, Mann–Whitney U test and chi-square test, as appropriate. Missing values on less than 20 % of items for each scale of all outcome measures will be replaced with the conditional mean values for the CBT or ST group.

#### Interrater reliability

Interrater reliability will be calculated by independent raters for SCID diagnoses before therapy starts (T1) and at the end of therapy, respectively after 26 hours of therapy (T2). We will also calculate interrater reliability for the treatment adherence. The percentage of agreement between the raters on SCID diagnoses, or treatment adherence, will indicate interrater reliability. Based on adequate total observer variance, we will also calculate a more robust measure (e.g. Cohen’s Kappa).

#### Therapist effects

One-way univariate ANOVA on primary outcome measures will show intra-group differences within both treatment conditions at the end of, and 9 and 12 months after the beginning of therapy. As there will be only eight CBT and ST therapists each, resulting in low power, the conclusion that each of them will be equally effective is tentative. Therefore, potential therapist effects will not be examined in this pilot study.

#### Clustering effects

CBT and ST will be administered in two independent centers. There will be considerable interaction among the therapists within each study arm, particularly during training and supervision. Within the ST condition, there will also be interaction between patients, above all in the group therapy session. Due to data dependency, we will conduct two-level (patients, treatment condition) linear regression analysis to account for positive intra-class correlations (ICCs) with respect to the primary outcome measures [91, 92].

#### Intention-to-treat and per-protocol analyses

The main analyses of all outcomes will be conducted on the intention-to-treat sample, comparing both treatment groups with 32 participants originally allocated after randomization [93]. This calculation will be regardless of whether the patients are detected to violate inclusion criteria, whether they complete therapy or not, or whether they are withdrawn from the study sample due to protocol deviations (e.g. changed use of medication, concurrent psychotherapy during the study period [94]). This will be followed by per-protocol analysis for the primary outcome measures (LSAS, SIAS, SPS) with only patients who fulfill all inclusion criteria, complete the intervention and do not deviate from the protocol.

#### Mixed-design ANOVA

We will perform mixed-design ANOVA to identify differences between treatment conditions [95]. Factors will be group (CBT or ST) and time (baseline, 8th, 15th, 20th therapy hour, end of therapy, respectively 26th therapy hour, 9 and 12 months after the beginning of therapy). Contrasts A will compare baseline with end of therapy. Contrasts B will indicate change between end of therapy, respectively 26th therapy hour, and 9 months after the beginning of therapy. Contrasts C will indicate change between end of therapy and 12 months after the beginning of therapy. We will control for differences in the length of treatment.

Time X Group interaction effect sizes will be assessed with partial eta-squared (η^2^) for the mixed-design ANOVA. Classification of effect sizes will be: η^2^ ≥ 0.01, small effect; η^2^ ≥ 0.06, medium effect; and η^2^ ≥ 0.14, large effect. Subsequently, for significant Time X Group interactions, simple effect analyses within and between groups will be performed [96]. Between-group effect sizes will be assessed with η^2^ and Cohen’s *d*. Classification of Cohen’s *d* will be: *d* ≥0.20, small effect; *d* ≥0.50, medium effect; and *d* ≥0.80, large effect [34]. In accordance with Coe [97], we will interpret Cohen’s *d* in terms of percentiles. Within-group effect sizes will be assessed with η^2^.

## Discussion

To the best of our knowledge, this will be the first prospective, open, interventive, balanced, bi-centric, explanatory pilot RCT comparing CBT, thoroughly evaluated for social anxiety disorders, and ST, a well-researched treatment approach that needs more evaluation with socially anxious patients. For study purposes, it is a challenge to make these two different approaches comparable in terms of manualization, duration of treatment, number of sessions, qualifications of therapists, institutional procedures, and allegiances of therapists and researchers. There are many differences that we will need to bridge. CBT is paid for by health insurances; ST is not. CBT training is state-regulated; ST training is regulated by professional associations. CBT is practiced almost exclusively by psychologists; ST by psychologists, social workers and other mental health professionals. CBT tends to use more sessions with shorter between-session intervals, and it does not intend to include important caregivers in treatment like ST does.

### Innovative aspects

This trial will be the first pilot study that directly compares CBT and ST, integrating a broad range of systemic methods, for social anxiety disorders. Former studies used either MST [6], an incremental design [5], PD, or did not focus on social anxiety disorders but mood and anxiety disorders in general [4]. The times of measurement and the instruments we will use are aligned with these three studies and the SOPHO-NET [48], allowing for direct comparisons of results.

### Biases and limitations

This trial is a pilot study and all statistical analyses will be descriptive and explorative, with the aim to obtain data that can then be used for planning a confirmatory RCT. Consequently, the main RCT is necessary before any confirmatory statement about the efficacy of ST for social anxiety disorders can reliably be made. It is also an open study, and patients and therapists will be informed about the study arm to which they will be allocated. Patients will present themselves to the study team, and no patient will be referred. The openness of this trial and type of recruitment is a naturalistic fact of “real world delivery of care” ([53]; p. 6). We try to balance direct and indirect allegiance by conducting a bi-centric trial, involving equally experienced ST and CBT experts, and implementing high methodological quality that appears to buffer allegiance [55]. The additional use of the OAT questionnaire [84] will help to test if we could balance allegiance. However, due to the small sample size, we will not perfectly control for allegiance effects.

### Perspectives of a confirmatory trial

We strive for a subsequent confirmatory multi-centric RCT comparing CBT and ST for social anxiety disorders. In addition to the psychological correlates we will assess in this pilot study, we hope to integrate biopsychological markers (e.g. alpha-amylase, cortisol, heart rate, heart rate variability).

### Trial status

The trial is ongoing and is currently recruiting.

## Abbreviations

ANOVA: analysis of variance
ASS: Adjustment to Symptomatology Scale
BAS: Burden Assessment Scale
BDI-II: Beck Depression Inventory II
BMI: body mass index
BSI: Brief Symptom Inventory
BST: brief strategic therapy
CBT: cognitive behavioral therapy
CGI: Clinical Global Impression
CI: confidence interval
CPP: Center for Psychological Psychotherapy
CSSRI-EU: Client Sociodemographic and Service Receipt Inventory
CTACS: Cognitive Therapy Adherence and Competence Scale
DAS-12: Dyadic Adjustment Scale
DPCCQ: Development of Psychotherapist Common Core Questionnaire
EXIS: Experience in Social Systems Questionnaire
GAF: Global Assessment of Functioning
HAM-A: Hamilton Anxiety Rating Scale
HIS: Helm Stierlin Institut
IIP: Inventory of Interpersonal Problems
IIP-32: Inventory of Interpersonal Problems
IMP: Institute for Medical Psychology
LSAS-SR: Liebowitz Social Anxiety Scale self-report measure
MAGST: Mannheim Society for Systemic Therapy
MST: minimal supportive therapy
OAT: Opinion About Treatment
PD: psychodynamic therapy
RCT: randomized controlled trial
ROCBT: resource-oriented cognitive-behavioral therapy
SACiP: Scale for the Multiperspective Assessment of General Change Mechanisms in Psychotherapy
SCID: Structured Clinical Interview according to the DSM
SCL-90-R: Symptom Checklist-90-R
SIAS: Social Interaction Anxiety Scale
SOPHO-NET: Social Phobia Psychotherapy Network
SPS: Social Phobia Scale
SRS: Session Rating Scale
ST: systemic therapy
STAS: Systemic Therapy Adherence Scale
URICA: University of Rhode Island Change Assessment Scale

## Footnotes

1 The term “bi-centric” is used with respect to the two centers involved in this study, the CPP and IMP.
